# Imbalance of TCA-related miRNA-mRNA networks involving IDH2, SDHA, SDHC, and SUCLG1 drives psoriasis development

**DOI:** 10.3389/fphys.2026.1884398

**Published:** 2026-07-14

**Authors:** Lin Chen, Zhan-zhong Qiao, Chang Xu, Ying Yao, Yuan-yuan Yang, Xin-yu Zhang, Shun-feng Cheng, Bin Zhang, Xue-yi Yang

**Affiliations:** 1 Department of Dermatology, The Second Affiliated Hospital of Henan University of Science and Technology, Luoyang, Henan, China; 2 College of Animal Science and Technology, Institute of Reproductive Sciences, Qingdao Agricultural University, Qingdao, China; 3 College of Life Science, Luoyang Normal University, Luoyang, China

**Keywords:** biomarkers, inflammation, metabolic reprogramming, plasma miRNA, psoriasis, TCA cycle

## Abstract

Psoriasis (PsO), a chronic inflammatory skin disorder, is characterized by keratinocyte hyperproliferation and immune dysregulation. Circulating microRNAs (miRNAs) have emerged as potential regulators of systemic metabolic and inflammatory pathways in PsO. In this study, we performed high-throughput sequencing of plasma miRNAs from PsO patients and healthy controls to explore their regulatory roles. Bioinformatic analysis identified several downregulated miRNAs, including hsa-miR-145-5p, hsa-miR-204-5p, hsa-miR-3913-5p, and hsa-miR-10a-5p, which were predicted to target key rate-limiting enzymes of the tricarboxylic acid (TCA) cycle (IDH2, SDHA, SDHC, SUCLG1). A miRNA-mRNA regulatory network was subsequently constructed to illustrate potential interactions between these miRNAs and their target mRNAs. Functional validation using miRNA inhibitors showed significant upregulation of IDH2, SDHA, SDHC, and SUCLG1 mRNA levels compared with the negative control (NC) group. These results suggest that downregulation of miRNAs may relieve the suppression of TCA cycle enzymes, leading to dysregulated glucose metabolism and contributing to the development of psoriasis. In conclusion, this study uncovers a novel mechanism by which miRNA downregulation mediates psoriasis via metabolic regulation and identifies potential targets for combined therapeutic strategies targeting both metabolism and miRNAs. However, the clinical translational relevance of these findings remains insufficiently clarified, and further studies are needed to determine their practical value in diagnosis, severity assessment, and therapeutic intervention.

## 1 Introduction

Psoriasis (PsO) is a chronic, immune-mediated inflammatory skin disorder affecting 2–4% of the global population. It is histologically characterized by keratinocyte hyperproliferation, abnormal differentiation, and a dense inflammatory infiltrate, reflecting the interplay between the epithelial and immune compartments (Mahe, 2016). Clinically, PsO imposes a substantial burden on patient quality of life and is associated with increased risks of psoriatic arthritis, cardiovascular disease, and metabolic syndrome (Grayson, 2012; Ji et al., 2021). Although the IL-23/IL-17A axis has been established as a central driver of psoriatic inflammation, the upstream molecular mechanisms that initiate and sustain the disease remain incompletely understood (Di Cesare et al., 2009; Orzan et al., 2025). Biologics targeting key inflammatory mediators have improved treatment outcomes; however, challenges such as variable responses, high costs, and long-term safety issues highlight the need for a deeper understanding of disease pathogenesis and the development of novel therapeutic strategies (Sbidian et al., 2022).

In recent years, biologic therapies targeting TNF-α, IL-12/23, IL-17A, IL-17 receptor, and IL-23 have substantially changed the treatment landscape of moderate-to-severe psoriasis (Wang et al., 2023; Strober et al., 2024). Among these agents, secukinumab, a fully human monoclonal antibody that selectively binds IL-17A, has shown strong clinical efficacy and a favorable safety profile in plaque psoriasis. IL-17A is a key effector cytokine in the IL-23/IL-17 immune axis and can induce keratinocyte-derived antimicrobial peptides, chemokines, and cytokines, thereby forming feed-forward inflammatory loops that amplify and sustain psoriatic inflammation. However, although IL-17A blockade has achieved significant therapeutic benefit, the immune response pathways and upstream molecular regulators involved in biologic treatment remain incompletely clarified. Recent transcriptomic and single-cell sequencing studies further suggest that psoriasis progression and treatment response are associated with complex immune-cell infiltration, keratinocyte differentiation, oxidative phosphorylation, reactive oxygen species-related pathways, and miRNA-mRNA regulatory networks (E et al., 2024). These findings indicate that miRNA-mediated regulation may provide complementary insight into psoriasis pathogenesis and therapeutic response in the era of biologic therapy.

Emerging evidence indicates that microRNAs (miRNAs), a class of small (~22–25 nucleotides) non-coding RNAs that mediate post-transcriptional gene silencing, play pivotal roles in regulating inflammatory signaling, keratinocyte proliferation, and differentiation (Bartel, 2004; Xiuli and Honglin, 2021; Jiang et al., 2023; Nemeth et al., 2024; Olejnik-Wojciechowska et al., 2024). Aberrant expression of miRNAs has been reported in psoriatic lesions, with functional consequences for disease progression. For example, miR-203 is consistently overexpressed and promotes inflammation by targeting SOCS-3, while miR-125b downregulation enhances keratinocyte proliferation through FGFR2 (Sonkoly et al., 2007; Xu et al., 2011). High-throughput sequencing has further uncovered previously unrecognized miRNAs dysregulated in psoriasis, expanding our understanding of the complex miRNA regulatory network (Srivastava et al., 2019; Abdallah et al., 2023).

Beyond immune dysregulation, metabolic reprogramming has emerged as a critical factor in psoriasis pathogenesis (Liu et al., 2023; Sarandi et al., 2023). While previous studies have largely focused on rate-limiting enzymes of fatty acid metabolism, the role of TCA cycle rate-limiting enzymes and their regulation by miRNAs remains poorly explored (Zhu et al., 2021; Cai et al., 2025). Key TCA enzymes can be directly regulated by miRNAs, influencing cellular energy metabolism, keratinocyte proliferation, and inflammatory responses (Liu et al., 2023). Dysregulation of this miRNA-mRNA-TCA axis may therefore contribute to disease progression; however, mechanistic insights into how miRNA-mediated control of TCA enzymes affects psoriasis pathogenesis are lacking.

In this study, we performed high-throughput profiling of plasma miRNAs from psoriasis patients and healthy controls and used bioinformatic predictions to identify miRNAs targeting TCA cycle rate-limiting enzymes. Functional validation through RT-qPCR and miRNA inhibitor assays confirmed the regulatory relationships between selected miRNAs and their enzyme targets. Our findings reveal a novel mechanism by which miRNA-mediated regulation of TCA cycle enzymes contributes to metabolic dysregulation and psoriasis development, highlighting potential therapeutic targets beyond the well-studied lipid metabolism pathways.

## 2 Material and methods

### 2.1 Patients and sample collection

Patients with psoriasis vulgaris and healthy controls were recruited from the outpatientdepartment of Dermatology at the Second Affiliated Hospital of Henan University of Science and Technology. Ethical approval was obtained from the hospital, and all participants provided written informed consent prior to sampling (Boehncke and Schon, 2015). Individuals with other immune or systemic diseases, or who had received immunosuppressive or local treatments before diagnosis, were excluded. The demographic and clinical characteristics of the enrolled patients, including PASI-based severity assessment, are summarized in **Supplementary Table 1**.

The collection of patients with psoriasis vulgaris and healthy control plasma samples has been previously described (Chen et al., 2021). In brief, fasting peripheral blood was collected, plasma was separated by centrifugation, and samples were stored at −80 °C for subsequent miRNA analysis. Skin tissue samples were collected from psoriasis lesions and corresponding healthy controls as previously described (Castillo et al., 2023). In brief, biopsy specimens were obtained under sterile conditions, immediately placed in ice-cold collection medium, and processed for downstream analyses, including RNA extraction and histological examination.

### 2.2 Hematoxylin-eosin staining

Skin tissue samples were first fixed in 4% paraformaldehyde (PFA; Sorlabio, P1110, Beijing, China) at 4 °C overnight. After fixation, the tissues were dehydrated through a graded ethanol series and embedded in paraffin. Serial sections of 7 μm thickness were prepared using a microtome. The sections were then deparaffinized, rehydrated, and stained with hematoxylin for 5 minutes, followed by rinsing twice with distilled water. After staining, the slides were mounted with neutral gum and visualized using a light microscope (Olympus BX51, Japan). Representative images were captured for subsequent morphological analysis.

### 2.3 Enzyme-linked immunosorbent assay

Plasma concentrations of IFN-γ, IL-17A, IL-17F, IL-23, and TET2 (**Supplementary Table 2**) were measured using commercially available ELISA kits according to the manufacturers’ instructions. Briefly, plasma samples and standards were added to pre-coated 96-well plates and incubated to allow antigen-antibody binding. After washing to remove unbound material, a horseradish peroxidase (HRP)-conjugated detection antibody was added, followed by substrate solution to develop color. The reaction was terminated with stop solution, and absorbance was measured at 450 nm using a microplate reader (Cytation1, BioTek, USA). All samples were measured in duplicate, and concentrations were calculated based on standard curves.

### 2.4 HaCaT cell culture

human immortalized keratinocytes (HaCaT) cells were purchased from HaiXing Bio (Beijing, China). Cells were cultured in complete medium provided by the supplier, consisting of Dulbecco’s Modified Eagle Medium (DMEM) supplemented with 10% fetal bovine serum (FBS) and 1% penicillin–streptomycin. Cultures were maintained at 37 °C in a humidified atmosphere containing 5% CO_2_ and subcultured when reaching 80–90% confluence.

### 2.5 RNA extraction and RT-qPCR

Total RNA was isolated from collected cells using standard extraction protocols, as describedpreviously (Qiao et al., 2024). Complementary DNA (cDNA) was then synthesized from the extracted RNA. Relative expression levels of target genes and miRNAs were quantified using the 2^−ΔΔCt^ method, with GAPDH and U6 serving as internal reference controls (**Supplementary Table 3**). All reactions were performed in technical triplicates to ensure reproducibility.

### 2.6 Western blot

Proteins were extracted from cells using RIPA lysis buffer (Beyotime, P0013C, Nantong, China)supplemented with SDS-PAGE loading buffer (Beyotime, P0015L, Nantong, China). The lysates were denatured by boiling at 95 °C for 5 minutes. Equal amounts of protein were separated on 10% SDS-PAGE gels with an initial run through the stacking gel at 80 V for 30 minutes, followed by electrophoresis through the resolving gel at 120 V for 90–130 minutes. Proteins were then transferred onto PVDF membranes (Millipore, ISEQ00010, USA) using a constant current of 200 mA for 150 minutes. Membranes were blocked in 5% BSA in TBST overnight at 4 °C, incubated with primary antibodies (**Supplementary Table 2**), washed, and then incubated with secondary antibodies (**Supplementary Table 2**) for 90 minutes at room temperature. Protein signals were visualized using a chemiluminescent detection system. GAPDH was used as the loading control, and densitometric analysis was performed using AlphaView SA software.

### 2.7 miRNA data analysis

Fasting peripheral blood samples were collected from psoriasis patients and age-, gender-, andBMI-matched healthy controls. Plasma was isolated by centrifugation and stored at −80 °C until further processing. Small RNA was extracted and subjected to high-throughput sequencing using the HiSeq 4000 platform (Novogene, Beijing, China). Raw sequencing reads were first assessed for quality and trimmed to remove adapters and low-quality reads using FastQC and fastp. Clean reads were then mapped to the human reference genome (GRCh38) using STAR, and miRNA expression levels were quantified (**Supplementary Table 4**). Differential expression analysis was performed using DESeq2 to identify significantly altered miRNAs (DEmiRNAs) between psoriasis patients and healthy controls (|log_2_FC| ≥ 0.58 and adjusted p-value (padj) < 0.05). Potential target mRNAs of DEmiRNAs were predicted using TargetScan and miRanda, followed by functional enrichment analysis with clusterProfiler. Gene Ontology (GO) and Kyoto Encyclopedia of Genes and Genomes (KEGG) results were visualized using Cytoscape, and a miRNA-mRNA regulatory network was constructed.

### 2.8 miRNA inhibitor transfection

miRNA inhibitors were delivered into cells using GP-Transfect-Mate reagent according to the manufacturer’s instructions (Qiao et al., 2024). Briefly, the inhibitor complexes were added to the culture medium, and cells were incubated under standard conditions. After 72 hours, cells were collected for downstream analyses, including RT-qPCR and functional assays.

### 2.9 Statistical analysis

All experiments were conducted with at least three biological replicates, and data are presented as mean ± standard deviation (SD). Statistical comparisons were performed using GraphPad Prism, and differences among groups were assessed by one-way analysis of variance (ANOVA), followed by Tukey’s *post-hoc* test for multiple comparisons. Significance levels were defined as *p < 0.05* (*), *p < 0.01* (**), and *p < 0.001* (***), whereas *p > 0.05* was considered not significant (ns).

## 3 Results

### 3.1 Elevated inflammatory cytokines in psoriasis plasma

**Figure 1:**
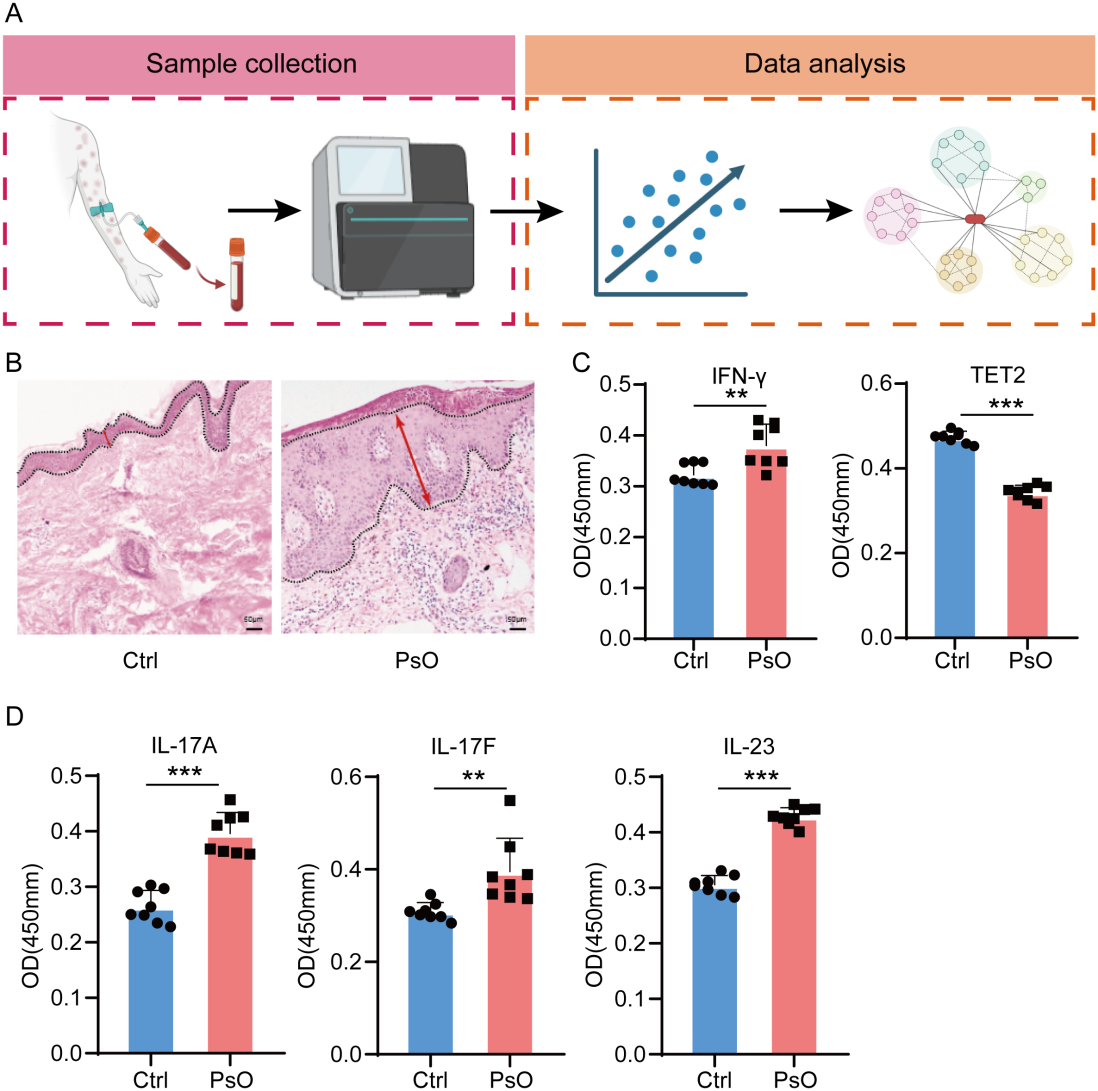
Elevated Inflammatory Cytokines in Psoriasis Plasma. **(A)** Schematic diagram of the research design. **(B)** Representative hematoxylin and eosin (HE) staining of skin tissue from healthy controls (Ctrl) and psoriasis patients (PsO). The red arrow indicates epidermal thickening in PsO lesions. Scale bar, 30 μm. **(C)** Plasma concentrations of IFN-γ and TET2 measured by ELISA. **(D)** Plasma concentrations of IL-17A, IL-17F, and IL-23 measured by ELISA. (All experiments contain at least 3 biological replicates, **p < 0.01, ***p < 0.001).

The overall experimental workflow is summarized in **Figure 1A**. To characterize the pathological features of PsO patients, we analyzed both histological changes and inflammatory factor expression. HE staining was performed on skin tissues from PsO patients and healthy controls (Ctrl) to assess histopathological characteristics. The results indicated that marked epidermal thickening was observed in psoriasis skin. In contrast, the control skin exhibited intact tissue architecture, normal epidermal thickness, and well-organized keratinocyte layers (**Figure 1B**). Plasma levels of inflammatory and regulatory factors were subsequently measured using ELISA. The results showed that IFN-γ, IL-17A, IL-17F, and IL-23 levels were significantly elevated in PsO patients, whereas TET2 levels were significantly decreased, compared with Ctrl (**Figures 1C, D**). To further validate the ELISA results, Western blot analysis of plasma IL-23 was performed (**Supplementary Figure 1A**), confirming the increased IL-23 protein levels in PsO patients. Additionally, PCNA expression in skin tissue was examined by Western blot (**Supplementary Figure 1B**). The results showed that PCNA levels were markedly higher in PsO lesions compared with control tissue, indicating enhanced keratinocyte proliferation in affected skin. Collectively, these observations demonstrate systemic inflammatory activation and local epidermal hyperproliferation in psoriasis patients (Hawkes et al., 2018; Kim et al., 2023; Jairath et al., 2024; Zhu et al., 2024).

### 3.2 Analysis of DEmiRNAs

**Figure 2:**
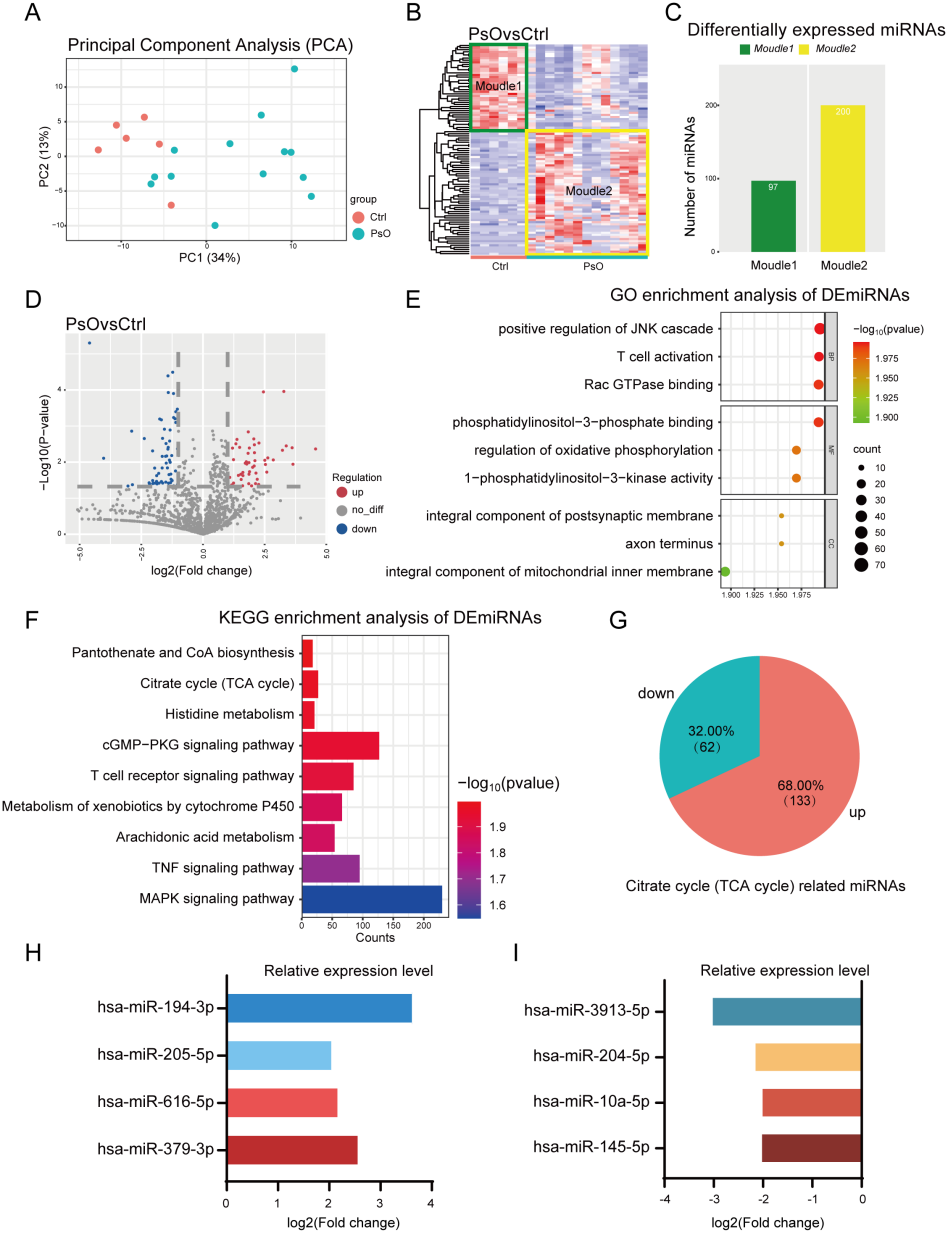
Analysis of DiEmRNAs. **(A)** Principal Component Analysis (PCA) of plasma miRNA expression. Psoriasis patients (PsO) and healthy controls (Ctrl) are clearly separated, indicating distinct miRNA expression profiles. **(B)** Heatmap showing hierarchical clustering of differentially expressed miRNAs. Two major modules (Module1 and Module2) are highlighted. **(C)** Number of miRNAs in each module. Module1 (green) and Module2 (yellow) represent differentially expressed miRNA clusters. **(D)** Volcano plot showing upregulated (red) and downregulated (blue) miRNAs in PsO plasma relative to controls. Gray dots represent miRNAs without significant differential expression. **(E)** Gene Ontology (GO) enrichment analysis of predicted targets of differentially expressed miRNAs. Biological Process (BP), Molecular Function (MF), and Cellular Component (CC) terms are shown. Dot color represents –log_10_(p-value), and dot size indicates the number of genes associated with each term. **(F)** KEGG pathway enrichment analysis of predicted targets of differentially expressed miRNAs. Bar height represents the number of target genes associated with each pathway, and color indicates –log_10_(p-value). Pathways related to metabolism and immune response, including the TCA cycle, are highlighted. **(G)** Proportion of TCA cycle-related miRNAs that were upregulated (red) or downregulated (blue) in PsO plasma. The pie chart shows percentages and number of miRNAs in each category. **(H, I)** Differential expression of selected miRNAs detected by high-throughput sequencing. **(H)** Upregulated miRNAs (hsa-miR-194-3p, hsa-miR-205-5p, hsa-miR-616-5p, hsa-miR-379-3p). **(I)** Downregulated miRNAs (hsa-miR-3913-5p, hsa-miR-204-5p, hsa-miR-10a-5p, hsa-miR-145-5p) in PsO plasma relative to healthy controls.

To investigate the systemic molecular alterations in psoriasis, plasma miRNA expression profiles were analyzed by high-throughput sequencing. Principal component analysis (PCA) demonstrated clear separation between PsO and Ctrl, indicating distinct miRNA expression patterns (**Figure 2A**). Hierarchical clustering and heatmap analysis revealed two major modules of differentially expressed miRNAs (Module1 and Module2) between the two groups (**Figure 2B**), with 97 miRNAs in Module1 and 200 miRNAs in Module2 (**Figure 2C**). Differential expression analysis identified numerous miRNAs that were significantly altered in PsO plasma compared with Ctrl. As shown in the volcano plot (**Figure 2D**), several miRNAs were upregulated, whereas others were downregulated, highlighting a global dysregulation of circulating miRNA networks in psoriasis. To explore the potential biological functions of the DEmiRNAs in PsO plasma, GO enrichment analysis was performed. The results indicated that target genes of DEmiRNAs were significantly enriched in processes related to T cell activation, positive regulation of the JNK cascade, Rac GTPase binding, phosphatidylinositol-3-phosphate binding, and regulation of oxidative phosphorylation (**Figure 2E**). KEGG pathway enrichment analysis further revealed that the predicted target genes were involved in multiple pathways, including the MAPK signaling pathway, TNF signaling pathway, T cell receptor signaling, cGMP-PKG signaling, and notably, the citrate cycle (TCA cycle), along with additional pathways such as pantothenate and CoA biosynthesis, histidine metabolism, arachidonic acid metabolism, and metabolism of xenobiotics by cytochrome P450 (**Figure 2F**). Among the TCA cycle-related miRNAs, 68% were upregulated and 32% were downregulated in PsO plasma, as shown in **Figure 2G**, indicating a predominant upregulation of miRNAs associated with key TCA cycle enzymes. These findings suggest that dysregulated miRNAs may contribute to metabolic reprogramming in psoriasis, potentially influencing energy metabolism and disease progression.

Based on the magnitude of expression changes, the most significantly altered TCA cycle-related miRNAs were identified and selected for display. Specifically, hsa-miR-194-3p, hsa-miR-205-5p, hsa-miR-616-5p, and hsa-miR-379-3p were upregulated, whereas hsa-miR-3913-5p, hsa-miR-204-5p, hsa-miR-10a-5p, and hsa-miR-145-5p were downregulated (**Figures 2H, I**). RT-qPCR analysis confirmed the sequencing results, showing that hsa-miR-194-3p, hsa-miR-205-5p, hsa-miR-616-5p, and hsa-miR-379-3p were significantly upregulated, whereas hsa-miR-145-5p, hsa-miR-204-5p, hsa-miR-3913-5p, and hsa-miR-10a-5p were significantly downregulated in PsO plasma compared with healthy controls (**Supplementary Figures 2A, B**).

### 3.3 Downregulated miRNAs and their regulatory network targeting TCA cycle enzymes in psoriasis

**Figure 3:**
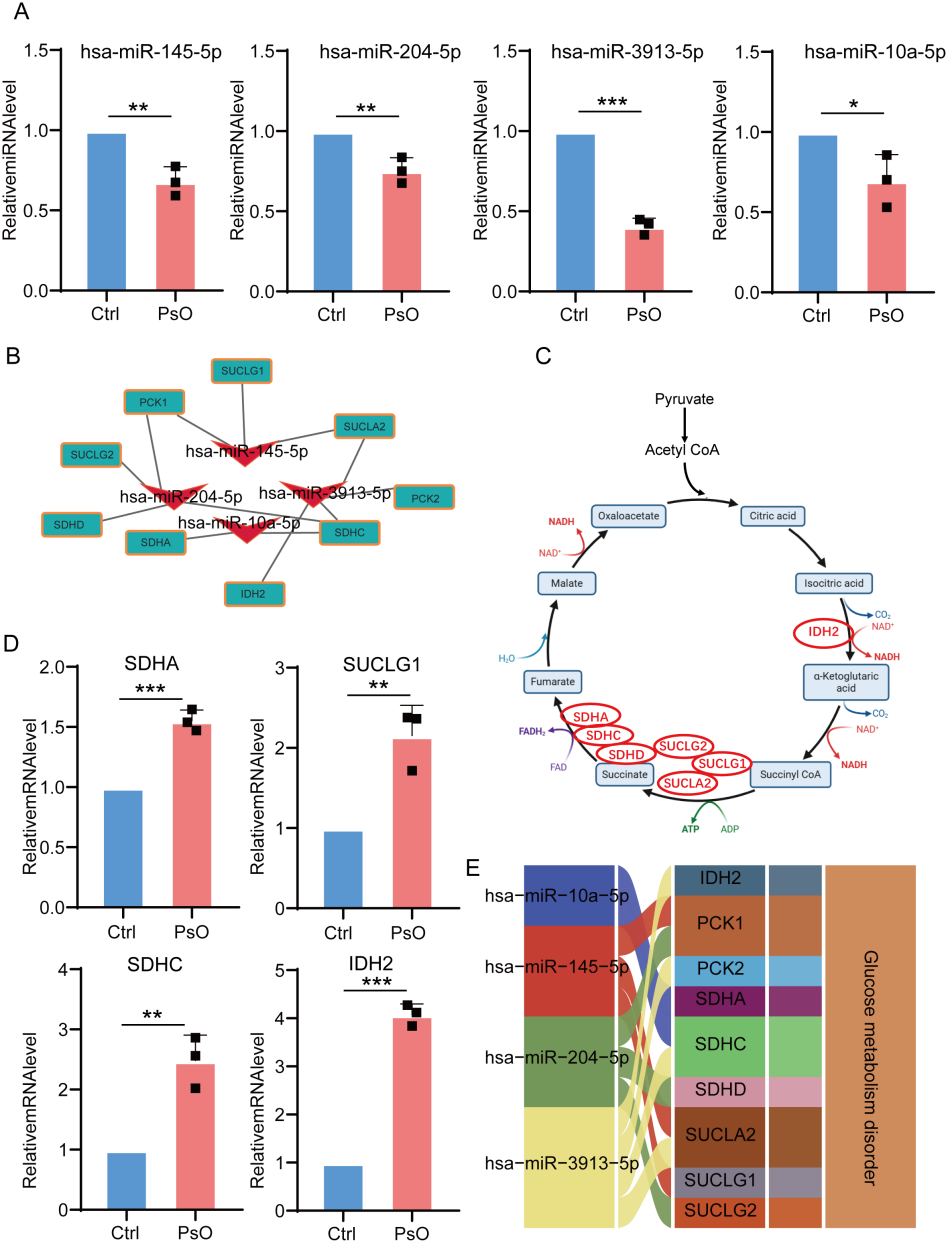
Downregulated miRNAs and Their Regulatory Network Targeting TCA Cycle Enzymes in Psoriasis. **(A)** RT-qPCR analysis of selected downregulated miRNAs in psoriatic skin tissue. Relative expression levels of hsa-miR-145-5p, hsa-miR-204-5p, hsa-miR-3913-5p, and hsa-miR-10a-5p were measured in PsO versus healthy controls (Ctrl). **(B)** Predicted miRNA-mRNA interaction network of downregulated miRNAs targeting TCA cycle rate-limiting enzymes. Red diamonds represent downregulated miRNAs; blue rectangles represent enzymes. Lines indicate predicted regulatory interactions. **(C)** Schematic representation of the TCA cycle highlighting the key rate-limiting enzymes targeted by downregulated miRNAs. Enzyme nodes regulated by miRNAs are indicated in red. **(D)** RT-qPCR validation of TCA cycle enzyme mRNA expression in PsO skin tissue. SDHA, SDHC, SUCLG1, and IDH2 mRNA levels were quantified. **(E)** Sankey diagram showing the relationships between downregulated miRNAs and their TCA cycle targets. The right column indicates the associated pathway of glucose metabolism disorder. (All experiments contain at least 3 biological replicates, *p < 0.05, **p < 0.01, ***p < 0.001).

We focused our analysis on the downregulated DEmiRNAs identified in plasma from PsO patients. To further investigate whether these altered miRNAs were also dysregulated in affected tissue, we measured their expression in psoriatic skin samples. The results demonstrated that the same miRNAs—hsa-miR-145-5p, hsa-miR-204-5p, hsa-miR-3913-5p, and hsa-miR-10a-5p—were also significantly downregulated in PsO skin, consistent with the plasma data (**Figure 3A**). To explore the potential regulatory targets of these downregulated miRNAs, a miRNA-mRNA interaction network was constructed (**Figure 3B**). The analysis revealed that these miRNAs potentially regulate multiple TCA cycle rate-limiting enzymes, including IDH2, SDHA, SDHC, SUCLG1, SUCLG2, SUCLA2, PCK1, and PCK2. To further illustrate the potential impact of these downregulated miRNAs on cellular metabolism, a schematic of the TCA cycle was generated highlighting the key rate-limiting enzymes targeted by the validated miRNAs (**Figure 3C**). The regulatory effects of these miRNAs were primarily concentrated on the steps controlling the conversion of succinyl-CoA to downstream intermediates, indicating that miRNA downregulation may affect energy production and metabolic flux in psoriasis. Further validation was performed to assess the expression of TCA cycle rate-limiting enzymes in PsO skin tissue. RT-qPCR analysis revealed that the mRNA levels of SDHA, SDHC, SUCLG1, and IDH2 were significantly elevated in PsO samples compared with controls (**Figure 3D**). These findings are consistent with the miRNA-mRNA network analysis (**Figures 3B, C**) and suggest that the downregulation of specific miRNAs may relieve suppression of multiple key TCA enzymes, potentially contributing to enhanced metabolic activity and energy production in psoriatic skin. To further explore the potential functional consequences of miRNA-mediated regulation, a Sankey diagram was generated to visualize the interactions between the validated downregulated miRNAs and their TCA cycle enzyme targets (**Figure 3E**).

### 3.4 Verification of miRNA-mRNA regulatory network

**Figure 4:**
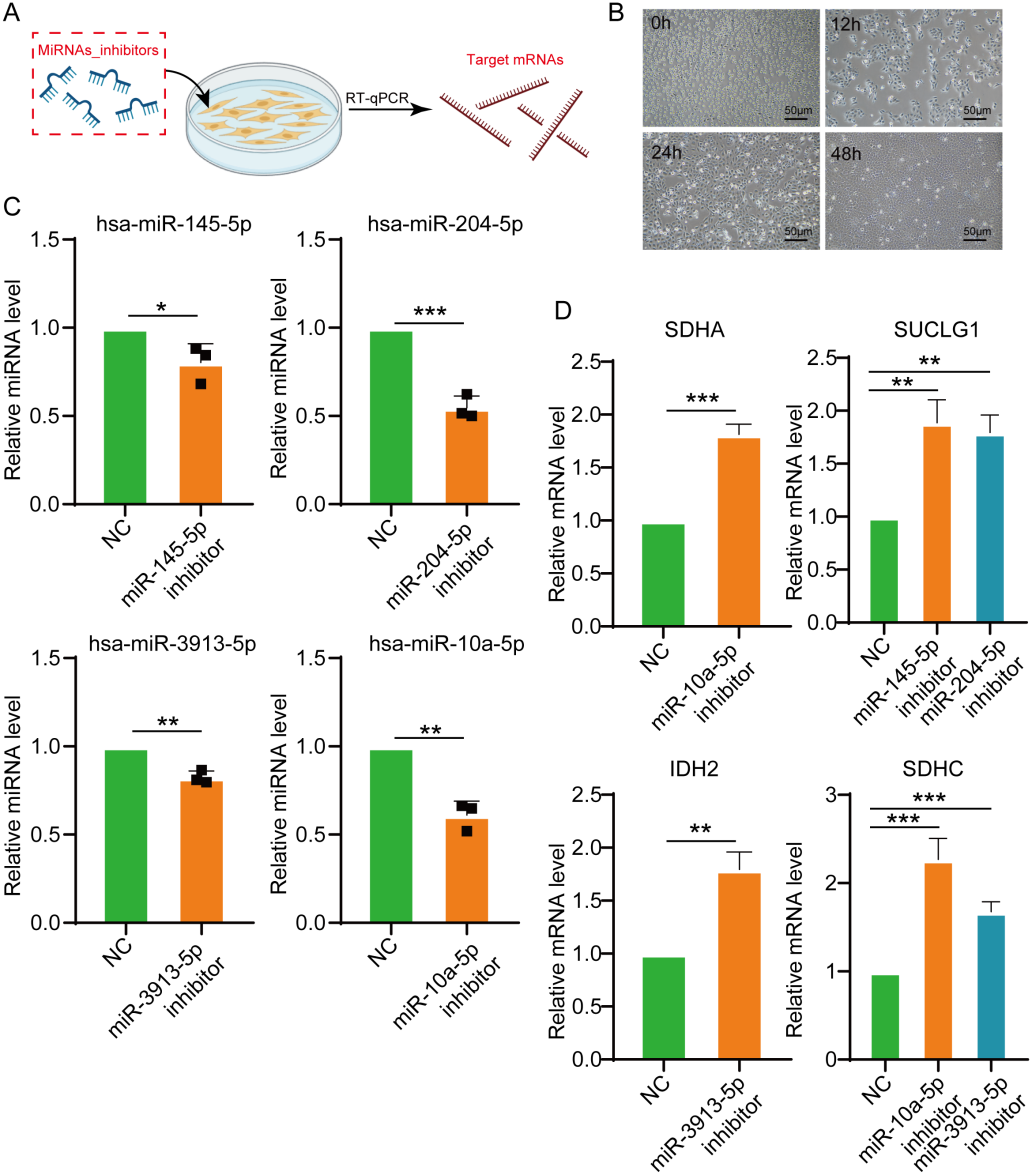
Verification of miRNA-mRNA regulatory network. **(A)** Schematic illustration of the miRNA inhibitor experiment. Specific downregulated miRNAs were inhibited in HaCaT cells, and target mRNA expression was measured by RT-qPCR 72 hours post-transfection. **(B)** Representative images of HaCaT cell morphology at 0, 12, 24, and 48 hours post-transfection. Scale bar: 50 µm. **(C)** RT-qPCR validation of miRNA inhibition. Relative expression levels of hsa-miR-145-5p, hsa-miR-204-5p, hsa-miR-3913-5p, and hsa-miR-10a-5p were significantly reduced in inhibitor-treated cells compared with negative control (NC). **(D)** RT-qPCR analysis of TCA cycle rate-limiting enzyme mRNA expression in HaCaT cells following miRNA inhibition. SDHA, SDHC, SUCLG1, and IDH2 were upregulated in response to inhibition of their corresponding miRNAs, confirming the predicted regulatory relationships. (All experiments contain at least 3 biological replicates, *p < 0.05, **p < 0.01, ***p < 0.001).

To validate the regulatory relationships predicted in the miRNA-mRNA network, miRNA inhibitor experiments were performed (**Figure 4A**). Selected downregulated miRNAs, including hsa-miR-145-5p, hsa-miR-204-5p, hsa-miR-3913-5p, and hsa-miR-10a-5p, were inhibited in HaCaT cells to assess their effects on the expression of target TCA cycle enzymes (**Figure 4B**). Cells were transfected with specific miRNA inhibitors, and RT-qPCR analysis was conducted 72 hours post-transfection to quantify changes in mRNA levels of predicted targets. RT-qPCR analysis confirmed effective inhibition, showing that the levels of all four miRNAs were significantly reduced in the inhibitor-treated group compared with the negative control (NC) (**Figure 4C**). Subsequently, the mRNA levels of TCA cycle rate-limiting enzymes were measured to determine the functional impact of miRNA inhibition. The results indicated that SDHA, SDHC, SUCLG1, and IDH2 mRNA levels were significantly increased in cells transfected with the corresponding miRNA inhibitors (**Figure 4D**). Combined inhibition of multiple miRNAs also led to an additive upregulation of target enzymes. These findings demonstrate that downregulated miRNAs directly regulate TCA cycle enzymes, and that relieving this repression results in enhanced expression of key metabolic genes, supporting their role in metabolic reprogramming in psoriasis.

**Figure 5:**
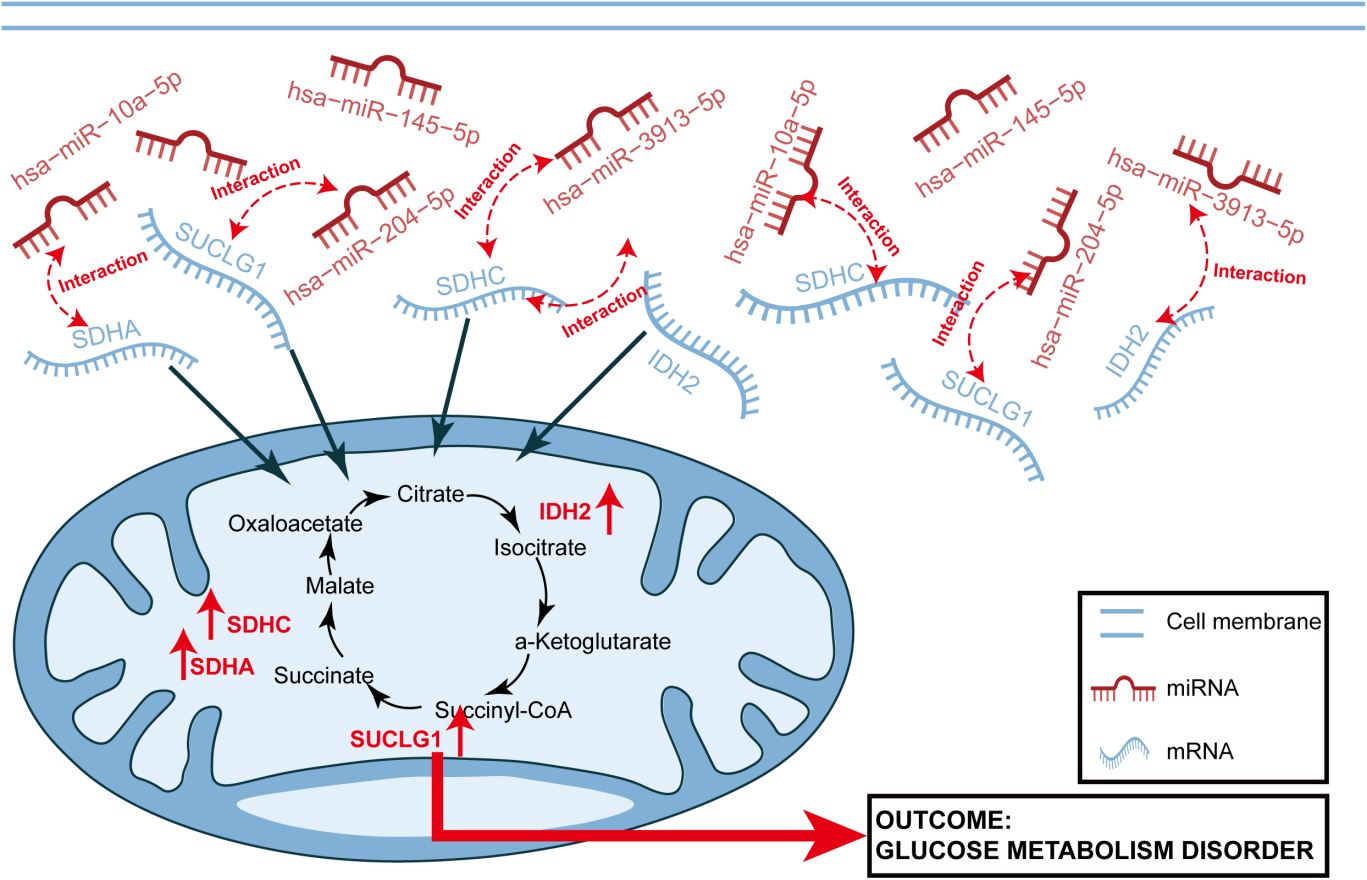
Molecular dynamics diagram.

Based on the integration of plasma and tissue miRNA expression, miRNA-mRNA network analysis, and functional validation experiments, a schematic model was constructed to summarize the regulatory mechanism (**Figure 5**). In this model, the downregulated miRNAs hsa-miR-145-5p, hsa-miR-204-5p, hsa-miR-3913-5p, and hsa-miR-10a-5p are predicted to target multiple TCA cycle rate-limiting enzymes, including IDH2, SDHA, SDHC, and SUCLG1. The suppression of these miRNAs is alleviated in psoriasis, resulting in upregulation of the corresponding enzymes. This upregulation may enhance TCA cycle activity and contribute to glucose metabolic dysregulation in psoriatic cells. In summary, these findings suggest that downregulation of specific miRNAs leads to upregulation of TCA cycle rate-limiting enzymes, resulting in glucose metabolic dysregulation and contributing to the pathogenesis of psoriasis.

## 4 Conclusion

In this study, we systematically profiled plasma miRNAs in psoriasis patients and identified a subset of downregulated miRNAs that were consistently reduced in both plasma and skin tissue. Functional validation demonstrated that these miRNAs directly regulate multiple TCA cycle rate-limiting enzymes, including IDH2, SDHA, SDHC, and SUCLG1, and that inhibition of these miRNAs leads to upregulation of the corresponding targets. Our findings highlight a coordinated miRNA-TCA regulatory axis that contributes to metabolic reprogramming and glucose dysregulation in psoriasis. Collectively, this study reveals a novel mechanistic link between miRNA dysregulation and TCA cycle metabolism, providing potential therapeutic targets for modulating metabolic pathways in psoriasis.

## 5 Discussion

In this study, we systematically investigated the role of downregulated miRNAs in psoriasis pathogenesis. Our results demonstrated that a subset of miRNAs—hsa-miR-145-5p, hsa-miR-204-5p, hsa-miR-3913-5p, and hsa-miR-10a-5p—was consistently downregulated in both plasma and psoriatic skin tissue, as confirmed by RT-qPCR (**Figure 3A**; **Supplementary Figure 2**). Functional network analysis indicated that these miRNAs potentially regulate multiple TCA cycle rate-limiting enzymes, including IDH2, SDHA, SDHC, and SUCLG1, which was further supported by mRNA expression measurements in PsO skin and HaCaT cells (**Figures 3D**, **4D**). Moreover, miRNA inhibitor experiments in HaCaT cells validated the predicted miRNA-mRNA regulatory relationships, demonstrating that suppression of these miRNAs leads to upregulation of TCA enzymes (**Figures 4C, D**). Collectively, these results establish a coordinated miRNA-TCA regulatory axis that contributes to metabolic dysregulation in psoriasis.

Psoriasis is a multifactorial disease involving both immune dysregulation and metabolic remodeling. While the IL-23/IL-17 axis has been widely recognized as a central driver of psoriatic inflammation, the potential contribution of metabolic alterations, particularly within the TCA cycle, has remained largely unexplored (Lei et al., 2025; Lin et al., 2025). Our findings suggest that systemic and tissue-specific downregulation of a subset of miRNAs may relieve repression on multiple TCA cycle rate-limiting enzymes, thereby enhancing metabolic flux and potentially sustaining keratinocyte hyperproliferation and inflammatory signaling. This provides a mechanistic link between post-transcriptional regulation by miRNAs and cellular energy metabolism in psoriasis.

Previous studies have predominantly focused on lipid metabolism and glycolytic reprogramming in psoriatic skin (Bochenska and Gabig-Ciminska, 2020; Nowowiejska et al., 2021; Tubau-Juni et al., 2021; Chen et al., 2023), while miRNA-mediated control of the TCA cycle has received little attention. By integrating plasma and tissue miRNA profiles, constructing miRNA-mRNA interaction networks, and performing functional validation in HaCaT cells, our study demonstrates that downregulated miRNAs coordinately regulate enzymes such as IDH2, SDHA, SDHC, and SUCLG1, which are essential for maintaining TCA cycle homeostasis. The observed upregulation of these enzymes following miRNA inhibition supports a model in which miRNA loss contributes to enhanced energy production, which may fuel both keratinocyte proliferation and local immune responses (Ruiz-Ponce et al., 2025; Hu et al., 2026).

Emerging evidence suggests that alterations in the TCA cycle play a significant role in psoriasis pathophysiology, although studies in this area remain limited (Liu et al., 2023; Sarandi et al., 2023). Recent studies indicate that key enzymes of the TCA cycle, such as IDH2 and SDH complexes, may influence cellular redox balance, reactive oxygen species (ROS) production, and ATP generation, all of which are critical for sustaining hyperproliferative keratinocytes and local inflammation (Kiguchi et al., 2020; Oluwole et al., 2026). Some reports have suggested that metabolic flux through the TCA cycle can modulate cytokine production and inflammatory signaling, highlighting a direct link between energy metabolism and immune dysregulation in psoriatic lesions (Guo et al., 2023; Sieminska et al., 2024). Despite these advances, miRNA-mediated regulation of TCA cycle enzymes in psoriasis has been largely unexplored. Our study extends this knowledge by demonstrating that downregulated miRNAs directly target multiple TCA cycle rate-limiting enzymes, and that inhibition of these miRNAs leads to enhanced enzyme expression and potential metabolic reprogramming.

Emerging evidence further supports a functional role of key TCA cycle enzymes, particularly IDH2 and SDH complexes, in regulating keratinocyte biology (Gambichler et al., 2018; Xu et al., 2020). IDH2-mediated control of mitochondrial redox homeostasis and NADPH production has been implicated in maintaining cellular energy balance, thereby influencing keratinocyte proliferation and survival under inflammatory conditions (Park et al., 2018). Similarly, SDH serves as a critical metabolic hub linking the TCA cycle and electron transport chain, and its dysregulation has been associated with altered reactive oxygen species (ROS) production and inflammatory signaling, both of which are key features of psoriatic epidermal hyperproliferation (Li et al., 2026). Importantly, recent studies have demonstrated that miRNAs can directly target these metabolic enzymes; for example, miR-145 and other miRNAs have been reported to regulate IDH2 and SDH expression, thereby modulating metabolic flux and inflammatory responses (Sharma et al., 2024). These observations are consistent with our findings that downregulated miRNAs relieve suppression of IDH2, SDHA, SDHC, and SUCLG1, suggesting a coordinated miRNA–TCA regulatory network that contributes to keratinocyte hyperproliferation and psoriasis progression.

From a clinical translational perspective, the miRNA-TCA regulatory axis identified in this study may serve as a potential biomarker for psoriasis stratification and therapeutic monitoring, because circulating miRNAs are stable and can be detected in plasma (Haschka et al., 2023). However, their diagnostic accuracy, correlation with PASI-based disease severity, and predictive value for treatment response require validation in larger clinical cohorts. In addition, dysregulated miRNAs may contribute to immune drift or immune deviation during psoriasis progression and biologic treatment. Psoriasis is mainly driven by the IL-23/Th17 axis, whereas atopic dermatitis is largely associated with Th2-skewed immunity, and a dynamic antagonistic balance exists between Th17/Th1 and Th2 immune responses. Strong blockade of one immune axis may shift this balance toward the opposite immune phenotype and lead to paradoxical inflammatory reactions (Qin et al., 2025). Because miRNAs can coordinately regulate immune and metabolic genes, altered miRNA expression may influence cytokine production, keratinocyte metabolism, and immune-cell activation, thereby affecting immune homeostasis and therapeutic responsiveness. Future longitudinal studies are required to determine whether these miRNAs can predict immune drift, treatment response, or disease recurrence.

Several limitations of the current study should be noted. First, HaCaT cells, while widely used as a keratinocyte model, do not fully recapitulate the complex multicellular environment of psoriatic skin, including immune-epithelial interactions. Second, although plasma miRNA levels reflect systemic changes, the specific contributions of local skin microenvironment factors remain to be clarified. Third, while TCA cycle enzyme expression was quantified, direct functional measurements of metabolic flux, ATP production, or reactive oxygen species were not performed, leaving a gap in understanding the downstream metabolic consequences. Future studies integrating metabolomics, single-cell transcriptomics, and spatial profiling will be essential to elucidate the full impact of miRNA-TCA regulation in psoriasis.

In conclusion, our study reveals that the downregulation of specific miRNAs in psoriasis leads to upregulation of multiple TCA cycle rate-limiting enzymes, contributing to metabolic reprogramming, enhanced energy production, and keratinocyte hyperproliferation. This coordinated miRNA-TCA regulatory axis provides a mechanistic link between post-transcriptional gene regulation and metabolic alterations in psoriatic lesions. These findings not only deepen our understanding of psoriasis pathogenesis but also identify potential therapeutic targets for modulating metabolic pathways, offering new avenues for intervention in this complex inflammatory disease.

## Data Availability

The original contributions presented in the study are publicly available. This data can be found here: OMIX, China National Center for Bioinformation / Beijing Institute of Genomics, Chinese Academy of Sciences (https://ngdc.cncb.ac.cn/omix accession number. OMIX018406).

## Supplementary material

Supplementary Figure 1 Western blot validation of inflammatory and proliferation markers in psoriasis patients. **(A)** IL-23 protein levels in plasma from psoriasis patients (PsO) and healthy controls (Ctrl). GAPDH was used as a loading control. (B) PCNA protein expression in psoriatic skin tissue and healthy controls. GAPDH was used as a loading control.

Supplementary Figure 2 RT-qPCR validation of differentially expressed miRNAs in plasma from psoriasis patients. **(A)** Relative expression levels of selected upregulated miRNAs (hsa-miR-194-3p, hsa-miR-205-5p, hsa-miR-616-5p, hsa-miR-379-3p) in PsO plasma compared with healthy controls (Ctrl). **(B)** Relative expression levels of selected downregulated miRNAs (hsa-miR-145-5p, hsa-miR-204-5p, hsa-miR-3913-5p, hsa-miR-10a-5p) in PsO plasma compared with Ctrl. (All experiments contain at least 3 biological replicates, **p* < 0.05, ***p* < 0.01, ****p* < 0.001).

Supplementary Table 1 Clinical information from subjects.

Supplementary Table 2 Antibodies and Reagents used in this study.

Supplementary Table 3 Primers used for quantitative RT-qPCR.

Supplementary Table 4 The miRNA expression matrices.
