# Supplementary figures and images for: Imbalance of TCA-related miRNA-mRNA networks involving IDH2, SDHA, SDHC, and SUCLG1 drives psoriasis development
Source: Front Physiol. 2026 Jul 14;17:1884398. doi: 10.3389/fphys.2026.1884398 (PMC13407281; doi:10.3389/fphys.2026.1884398)

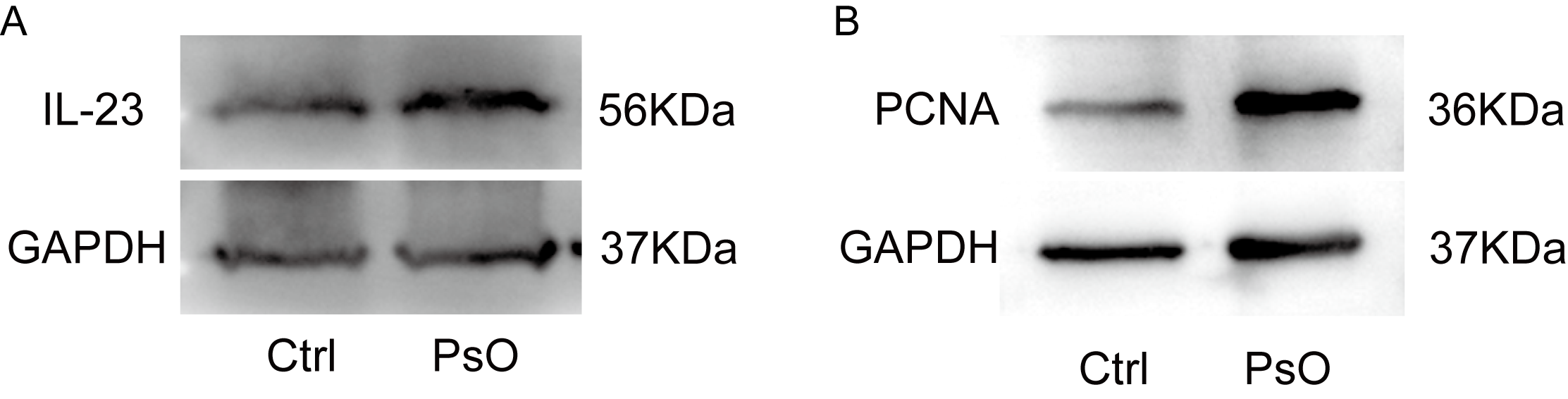

Supplement: Supplementary Figure 1 — Western blot validation of inflammatory and proliferation markers in psoriasis patients. (A) IL-23 protein levels in plasma from psoriasis patients (PsO) and healthy controls (Ctrl). GAPDH was used as a loading control. (B) PCNA protein expression in psoriatic skin tissue and healthy controls. GAPDH was used as a loading control. [file Image1.tif]

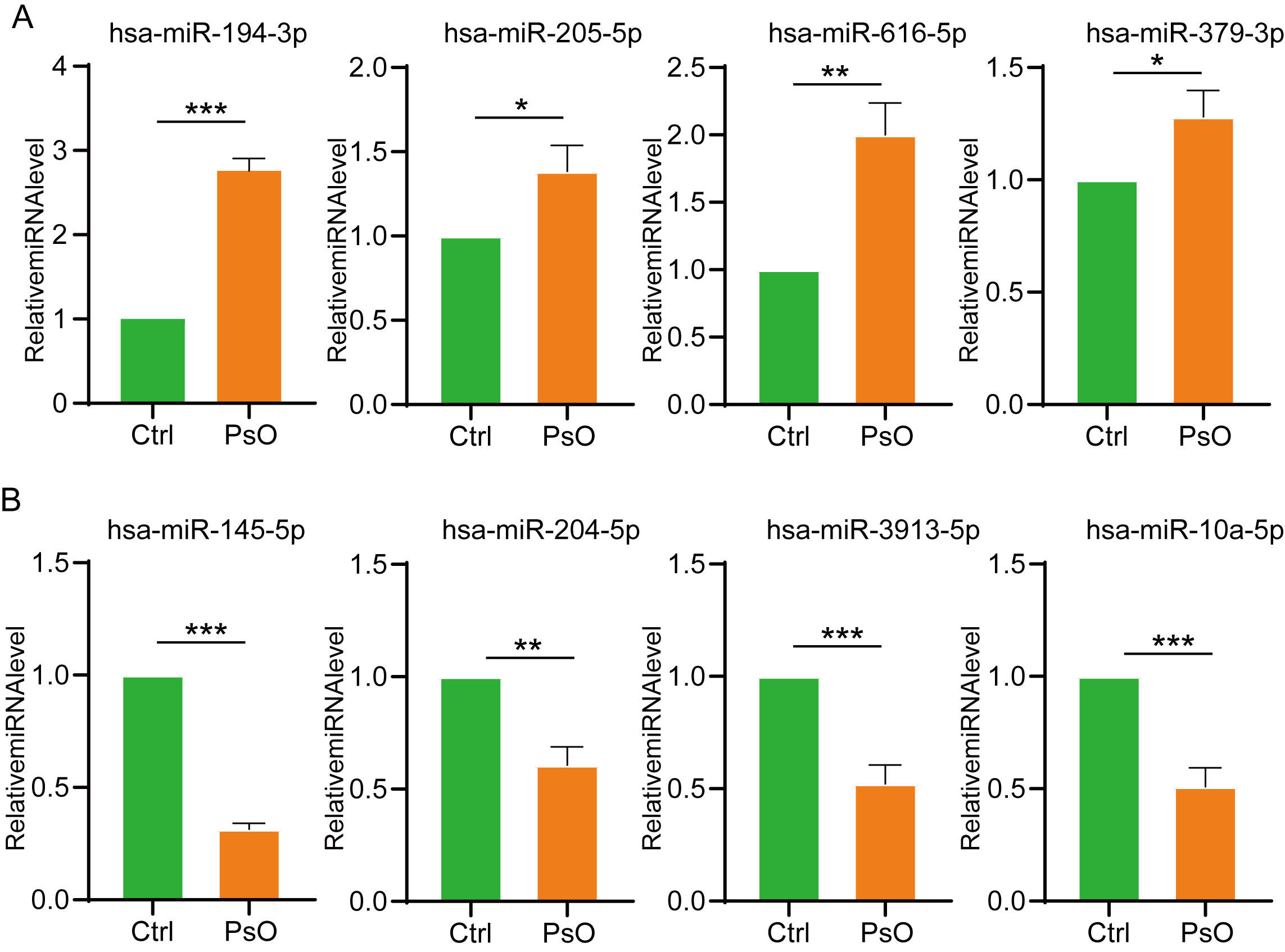

Supplement: Supplementary Figure 2 — RT-qPCR validation of differentially expressed miRNAs in plasma from psoriasis patients. (A) Relative expression levels of selected upregulated miRNAs (hsa-miR-194-3p, hsa-miR-205-5p, hsa-miR-616-5p, hsa-miR-379-3p) in PsO plasma compared with healthy controls (Ctrl). (B) Relative expression levels of selected downregulated miRNAs (hsa-miR-145-5p, hsa-miR-204-5p, hsa-miR-3913-5p, hsa-miR-10a-5p) in PsO plasma compared with Ctrl. (All experiments contain at least 3 biological replicates, *p < 0.05, **p < 0.01, ***p < 0.001). [file Image2.tif]
