# Supplementary material for: Imbalance of TCA-related miRNA-mRNA networks involving IDH2, SDHA, SDHC, and SUCLG1 drives psoriasis development
Source: Front Physiol. 2026 Jul 14;17:1884398. doi: 10.3389/fphys.2026.1884398 (PMC13407281; doi:10.3389/fphys.2026.1884398)
Supplement: Supplementary Table 1 — Clinical information from subjects. [file Table1.doc]

**Clinical information from subjects**

| Parameters | Con Group |  | Pso Group |
| --- | --- | --- | --- |
|  | N=85 |  | N=70 |
| Age, years, mean ± SD | 39.06 ± 15.02 |  | 41.03 ± 13.68*^ns/-^* |
| Male, n (%) | 39 (45.88%) |  | 31 (44.29%)*^ns/-^* |
| BMI, kg/m²,mean ± SD | 24.68 ± 4.38 |  | 23.00 ± 5.27*^ns/-^* |
| Disease duration, years, mean ± SD |  |  | 13.33 ± 6.31 |
| Medication time, years, mean ± SD |  |  |  |
| PASI |  |  | 18.25 ± 1.71 |

*a/a*：statistically significant vs con group/statistically significant vs pso group, *ns*: not significant, *** *p* ≤ 0.001.
