# Supplementary material for: Imbalance of TCA-related miRNA-mRNA networks involving IDH2, SDHA, SDHC, and SUCLG1 drives psoriasis development
Source: Front Physiol. 2026 Jul 14;17:1884398. doi: 10.3389/fphys.2026.1884398 (PMC13407281; doi:10.3389/fphys.2026.1884398)
Supplement: Supplementary Table 2 — Antibodies and Reagents used in this study. [file Table2.docx]

**Antibodies used in this paper**

| **Primary antibodies** | **Vendor** | **Dilution** | **Source** |
| --- | --- | --- | --- |
| GAPDH (WB) | Affnity (AF7021) | 1:1000 | Rabbit |
| PCNA(WB) | Abcam (ab29) | 1:1000 | Mouse |
| IL-23(WB) | Zenbio (163811) | 1:1000 | Rabbit |
| **Secondary antibodies** | **Vendor** | **Dilution** | **Source** |
| HRP-conjugated goat anti-rabbit IgG (WB) | Beyotime (A0258) | 1:1000 | Goat |
| HRP-conjugated donkey anti- goat IgG (WB) | Beyotime (A0181) | 1:1000 | Donkey |
| **Reagent** | **Vendor** | **Dilution** | **Source** |
| miRNA First-strand Synthesis kit  SPARK easy Fresh/Frozen Whole Blood Total RNA Extraction Kit  2×SYBR Green Qpcr Mix  SPARK script II RT Plus Kit | TaKaRa (638313)  SparkJade (AC0902)  SparkJade (AH0104)  SparkJade (AG0304) |  | Human  Human  Human  Human |
| IFN-γ (ELISA) | FANKEW (F0033-B) |  | Human |
| IL-17A (ELISA) | FANKEW (F9825-B) |  | Human |
| IL-17F(ELISA) | FANKEW (F10298-B) |  | Human |
| IL-23 (ELISA) | FANKEW (F0194-B) |  | Human |
| TET2(ELISA) | FANKEW (F0057-HB) |  | Human |
