# Supplementary material for: Imbalance of TCA-related miRNA-mRNA networks involving IDH2, SDHA, SDHC, and SUCLG1 drives psoriasis development
Source: Front Physiol. 2026 Jul 14;17:1884398. doi: 10.3389/fphys.2026.1884398 (PMC13407281; doi:10.3389/fphys.2026.1884398)
Supplement: Supplementary Table 3 — Primers used for quantitative RT-qPCR. [file Table3.docx]

**Primers used for quantitative RT-qPCR**

| *SUCLG1-F* | CTTTGTGCGTTGGCATTGGA |
| --- | --- |
| *SUCLG1-R* | GCCTTCTGTGGCAGAATCGT |
| *SDHA-F* | ACTGTTGCAGCACAGCTAGA |
| *SDHA-R* | GCTCTGTCCACCAAATGCAC |
| *SDHC-F* | TCAGCTCTGTATCAGAAATGCTGT |
| *SDHC-R* | CCTAGGTCCCACATCTGCAC |
| *IDH2-F* | TGGTGATGTTTCTGATGCCGA |
| *IDH2-R* | TCAGTCTGGTCACGGTTTGG |
| *GAPDH-F* | AATGGGCAGCCGTTAGGAAA |
| *GAPDH-R* | GCCCAATACGACCAAATCAGAG |
| *hsa-miR-145-5p-F (stem-loop)* | GTCCAGTTTTCCCAGGAATCCCT |
| *hsa-miR-145-5p-R* |  |
| *hsa-miR-204-5p-F (stem-loop)* | TTCCCTTTGTCATCCTATGCCT |
| *hsa-miR-204-5p-R* |  |
| *hsa-miR-3913-5p-F (stem-loop)* | TTTGGGACTGATCTTGATGTCT |
| *hsa-miR-3913-5p-R*  *hsa-miR-10a-5p-F (stem-loop)*  *hsa-miR-10a-5p-R*  *hsa-miR-194-3p-F (stem-loop)*  *hsa-miR-194-3p-R*  *hsa-miR-205-5p-F (stem-loop)*  *hsa-miR-205-5p-R*  *hsa-miR-616-5p-F (stem-loop)*  *hsa-miR-616-5p-F*  *hsa-miR-379-3p-F (stem-loop)*  *hsa-miR-379-3p-R*  miR-145-5p-inhibitor  miR-204-5p-inhibitor  miR-3913-5p-inhibitor  miR-10a-5p-inhibitor | TACCCTGTAGATCCGAATTTGTG  CCAGTGGGGCTGCTGTTATCTG  TCCTTCATTCCACCGGAGTCTG  ACTCAAAACCCTTCAGTGACTT  TATGTAACATGGTCCACTAACT  AGGGATTCCGGGAAAACTGGAC  AGGCATAGGATGACAAAGGGAA  ACATCAAGATCAGTCCCAA  CACAAATTCGGATCTACAGGGTA |

Table S3 Primers used for quantitative RT-qPCR F: Forward; R: Reverse
